# Exenatide and Dapagliflozin Combination Enhances Sertoli Cell Secretion of Key Metabolites for Spermatogenesis

**DOI:** 10.3390/biomedicines10051115

**Published:** 2022-05-11

**Authors:** João C. Ribeiro, Ana D. Martins, Ivana Jarak, Rui A. Carvalho, Marco G. Alves, Pedro F. Oliveira

**Affiliations:** 1 Departamento de Anatomia, UMIB—Unidade Multidisciplinar de Investigação Biomédica, ICBAS—Instituto de Ciências Biomédicas Abel Salazar, Universidade do Porto, 4050-313 Porto, Portugal; up202009612@up.pt (J.C.R.); anacdmartins@gmail.com (A.D.M.); alvesmarc@gmail.com (M.G.A.); 2 Laboratory for Integrative and Translational Research in Population Health (ITR), University of Porto, 4050-600 Porto, Portugal; 3 LAQV, Department of Chemistry, University of Aveiro, 3810-193 Aveiro, Portugal; 4 Department of Pharmaceutical Technology, Faculty of Pharmacy, Azinhaga de Santa Comba, University of Coimbra, Pólo III-Pólo das Ciências da Saúde, 3000-548 Coimbra, Portugal; jarak.ivana@gmail.com; 5 Department of Life Sciences, Faculty of Sciences and Technology, University of Coimbra, Calçada Martim de Freitas, 3000-456 Coimbra, Portugal; rac@uc.pt; 6 Nanoplatforms for Life, LAQV, REQUIMTE, Department of Pharmaceutical Technology, Faculty of Pharmacy, University of Coimbra, 3000-548 Coimbra, Portugal; 7 Biotechnology of Animal and Human Reproduction (TechnoSperm), Institute of Food and Agricultural Technology, University of Girona, 17003 Girona, Spain; 8 Unit of Cell Biology, Department of Biology, Faculty of Sciences, University of Girona, 17003 Girona, Spain

**Keywords:** diabetes mellitus, Sertoli cells, dapagliflozin, exenatide, spermatogenesis

## Abstract

The incidence of metabolic diseases such as type 2 diabetes mellitus (DM) and obesity has been increasing dramatically. Both diseases are closely linked and new approaches for type 2 DM treatment aim to enable weight loss. A combined therapy of dapagliflozin and exenatide has been used against type 2 DM, influencing allbody glucose dynamics. Spermatogenesis is highly dependent on the metabolic cooperation established between Sertoli cells (SCs) and developing germ cells. To study the effects of dapagliflozin and exenatide on SC metabolism, mouse SCs were treated in the presence of sub-pharmacologic, pharmacologic, and supra-pharmacologic concentrations of dapagliflozin (50, 500, 5000 nM, respectively) and/or exenatide (2.5, 25, 250 pM, respectively). Cytotoxicity of these compounds was evaluated and the glycolytic profile, glycogen content assay, and lipid accumulation of SCs were determined. Dapagliflozin treatment decreased fat cellular deposits, demonstrating its anti-obesity properties at the cellular level. Polytherapy of exenatide plus dapagliflozin increased lactate production by SCs, which has been reported to improve sperm production and quality. Thus, the results herein suggest that the use of these two pharmacological agents can protect male fertility, while improving their glucose homeostasis and inducing weight loss.

## 1. Introduction

Diabetes mellitus (DM) is a chronic illness that has reached pandemic proportions. Patients with this condition require continuous healthcare for not only glycemia control but also for reduction in the risk of developing comorbidities [1]. As an example, most patients with the most predominant type of DM (type 2 DM) also have a weight dysregulation prior to the development of chronic hyperglycemia [2]. This happens due to the linkage between overweight and insulin resistance. Hence, weight loss should be the main therapeutic measure to control glycemia in overweight type 2 DM individuals even though it is the most challenging aspect of the treatment [3]. Thus, anti-diabetic drugs with anti-obesogenic properties have been developed to improve the health and lifestyle of these patients. Two examples are the low-affinity/high-capacity sodium–glucose cotransporter 2 (SGLT2) inhibitors (e.g., dapagliflozin) and glucagon-like peptide (GLP)-1 receptor agonist (e.g., exenatide) [4]. SGLT2 is a glucose–sodium transporter with a crucial role in glucose reabsorption in the kidneys. SGLT2 expression was found to be insulin-dependent [5] and, in diabetic patients, tends to be increased [6]. This results in an increased threshold of glucose reabsorption in the kidneys of diabetic patients, further increasing glucose in circulation. SGLT2 inhibition by dapagliflozin is known to decrease caloric uptake and, thus, directly decrease glycemic levels and body weight [7,8]. On the other hand, GLP-1 is an incretin released after nutrient ingestion. Exenatide acts as an agonist to the GLP-1 receptor on the insulin-producing pancreatic β-cells by activating the insulin effector protein kinase A [9], which ultimately results in insulin production and secretion [9]. Exenatide has shown to decrease glycemia and decrease body weight in diabetic patients by increasing insulin secretion and decreasing satiety [7].

Moreover, multiple studies have already shown the effect of combined treatment of these drugs on glycemic control and weight loss with very optimistic results [10,11,12].

The incidence of type 2 DM is increasing among younger group ages [4] and some authors relate that fact with decreasing fertility rates [13]. In fact, proper metabolic cooperation between Sertoli cells (SCs) and developing germ cells are essential for male reproductive function [14]. However, this cooperation is impaired in diabetic and/or obese men [15]. Other anti-diabetic drugs, such as metformin and pioglitazone, and their effects on SC metabolism have already been described, showing the potential to promote male fertility by increasing lactate secretion by SCs [16,17]. Thus, we hypothesized that dapagliflozin and exenatide, alone or in combination, may alter SCs’ glucose metabolism. This work aims to decipher if such interventions can disrupt or promote the secretion of key metabolites for spermatogenesis by SCs.

## 2. Materials and Methods

### 2.1. Chemicals

Dapagliflozin and exenatide were purchased from Santa Cruz Biotechnology (Dallas, TX, USA). Fetal bovine serum (FBS) was purchased from Biochorm AG (Berlin, Germany). Insulin–transferrin–sodium selenite (ITS) supplement and gentamicin were obtained from Life Technologies (Carlsbad, CA, USA). Testosterone was purchased from Honeywell Fluka (Charlotte, NC, USA). Sulforhodamine B (SRB) was purchased from Biotum (Hayward, CA, USA). 3-(4,5-dimethylthiazol-2-yl)-2,5-diphenyl-2H-tetrazolium bromide (MTT) was obtained from Amresco (Parkway Solon, OH, USA). All reagents for polymerase chain reaction (PCR) were purchased from NZYTech (Lisbon, Portugal), including: NZY M-MuLV Reverse Transcriptase, random hexamer primers, NZTaq 2x Green Master Mix, dNTPs, reaction buffer, and Greensafe. The lactate dehydrogenase (LDH)-Cytox^TM^ Assay Kit was purchased from BioLegend (San Diego, CL, USA). The bicinchoninic acid (BCA) protein assay kit was purchased from Thermo Scientific (Waltham, MA, USA). Tetraethylbenzimidazolylcarbocyanine iodide (JC-1) dye was purchased from Life Technologies (Gaithersburg, MD, USA). Oil Red O staining and other chemicals were all purchased from Sigma-Aldrich (St. Louis, MO, USA) unless stated otherwise.

### 2.2. Sertoli Cell Culture

A Sertoli cell line (TM4) was purchased from ATCC (Manassas, VA, USA). TM4 SCs were seeded and grown in 75 cm^2^ T-flasks. Cells were incubated and maintained with SC culture medium containing Dulbecco’s modified Eagle medium and Ham’s nutrient mixture F12 (DMEM, Ham’s F12) in a proportion of 1:1, 15 mM of 4-(2-hydroxyethyl)-1-piperazineethanesulfonic acid (HEPES), 50 U/mL of penicillin, 50 mg/mL of streptomycin sulfate, 0.5 mg/mL of fungizone, 50 mg/mL of gentamicin, and 10% FBS. The medium was also supplemented with glucose to reach a concentration of 15.3 mM to mimic a hyperglycemic environment. The cells were handled in a laminar flow chamber and incubated at 37 °C with a 5% CO_2_ humified atmosphere.

### 2.3. Experimental Groups

To evaluate the potential effect of exenatide and dapagliflozin on TM4 SC physiology and metabolism, 10 experimental groups were designed. Cells were seeded and allowed to grow until 70–80% confluence in 10 cm tissue culture Petri dishes. Then, cells were treated without (control group) or with sub-pharmacologic, pharmacologic, and supra-pharmacologic concentrations of dapagliflozin (50 nM, 500 nM, and 5000 nM, respectively) or exenatide (2.5 pM, 25 pM, and 250 pM, respectively) for 24 h. These concentrations were chosen considering the dapagliflozin and exenatide pharmacological concentrations described in plasma of individuals under treatment [18,19]. For the study of the effects of combined therapy, the pharmacological concentrations of dapagliflozin (500 nM) and exenatide (25 pM) were added simultaneously in the medium. Treatment medium was similar to the Sertoli cell culture medium but without FBS supplementation. However, it was supplemented with ITS (insulin 10 µg/mL; transferrin 0.55 µg/mL; sodium selenite 0.0067 µg/mL), 100 U/L follicle-stimulating hormone (FSH), and 5 µmol/L testosterone. After the incubation period, extracellular media were collected for posterior analysis by proton nuclear magnetic resonance (^1^H-NMR) and the cells were removed from the plates with a trypsin–ethylenediamine tetraacetic acid (EDTA) solution. After detachment, the cells were counted with a Neubauer chamber and stored at −80 °C for protein extraction and glycogen quantification.

### 2.4. Cell Proliferation Assay

TM4 SC proliferation when treated with exenatide and/or dapagliflozin was assessed by SRB assay [20]. In brief, cells cultured in a 24-well plate were left to grow until reaching 60–70% confluence. After the 24 h incubation with the different treatments, the cells were fixed with a solution of 1% acetic acid in 99% methanol for at least 1 h at −20 °C. Then, cells were incubated with a solution of SRB at 0.05% prepared with 1% acetic acid in water, for 1 h at 37 °C. After washing the cells, SRB dye was removed from the cells with a 10 mM Tris solution with pH 10 and optical density was determined at 490 nm using the BioTek Synergy HT (BioTek, Winooski, VT, USA).

### 2.5. MTT Assay

TM4 SC viability was assessed by the MTT assay [21]. Cells were seeded and treated for 24 h in a 48-well plate. When 80–90% confluence was achieved, the treatment medium was replaced with 500 µL Sertoli cell medium and supplemented with 50 µL of MTT solution at 5 mg/mL. Cells were then incubated for 3 h at 37 °C and protected from light. After incubation, the medium was removed and the MTT crystals were dissolved by the addition of 250 µL of dimethyl sulfoxide (DMSO). Then, 100 µL of the solubilized crystal was transferred to a 96-well plate. The absorbance was measured at 570 nm and 655 nm in a BioTek Synergy HT (BioTek, Winooski, VT, USA) microplate multireader. The resulting values were divided by the mean of the control group and expressed in fold variation versus the control group.

### 2.6. Reverse Transcriptase Polymerase Chain Reaction

The tRNA obtained from TM4 SCs and control samples (testis, kidney, and lung tissue) were reversely transcribed in a mixture containing 1 µg of the samples’ tRNA, 1 µL of dNTPs (10 mM), and 2.5 µL of random hexamer primers (50 ng/µL), and sterile water was added until a final volume of 17 µL. The mixture was initially incubated for 5 min at 65 °C. After this, 1 µL of reverse transcriptase enzyme and 2 µL of the reaction buffer were added and incubated at 3 different temperatures for various durations: 25 °C for 10 min, 37 °C for 50 min, and 70 °C for 15 min. The resulting cDNA was used with exon–exon spanning primer sets to amplify SGLT2 and GLP-1 receptor cDNA fragments. PCR was performed using 1 µL of cDNA in 12.5 µL of the final volume solution of 6.25 µL of NZYTaq Green Master Mix, 1 µM of primer solution, and sterile water. The optimal cycle number and annealing temperature required for exponential amplification were optimized for each primer using standard procedures (Table 1). Then, samples were run in 1% agarose gel electrophoresis with GreenSafe (2 µL/200 mL), for approximately 30 min at 120 V. Bio-Rad ChemiDoc XRS (Bio-Rad, Hemel Hempstead, UK) was used to visualize the agarose gel.

### 2.7. Protein Extraction

TM4 SC total protein was isolated using radioimmunoprecipitation assay (RIPA) buffer formed with 0.5% sodium deoxycholate, 0.1% sodium dodecyl sulfate in phosphate-buffered saline (PBS). RIPA buffer was supplemented with 1% protease inhibitors cocktail and sodium orthovanadate at 10 mM. Fifty microliters of RIPA buffer were added to the cells pellet and subjected to agitation at 4 °C for 20 min. Then, samples were centrifuged for 20 min at 14,000× *g* at 4 °C. A Pierce^TM^ Microplate BCA Protein Assay Kit was used for protein quantification according to the manufacturer’s instructions. Different bovine serum albumin concentrations were used as standards for calibration and absorbance was measured at 560 nm using a BioTek Synergy HT (BioTek, Winooski, VT, USA) microplate multireader.

### 2.8. Intracellular Lactate Dehydrogenase Activity Assay

Intracellular lactate dehydrogenase activity was determined using the commercial LDH-Cytox^TM^ Assay Kit, following the instructions from the manufacturer. Briefly, 5 µg of freshly extracted protein was diluted in PBS to 50 µL. Then, the LDH assay substrate was added to all samples and left in a dark environment at 37 °C for 10 min. After this, the STOP solution was added and the absorbance of all the samples was measured at 490 nm using the BioTek Synergy HT (BioTek, Winooski, VT, USA) microplate reader.

### 2.9. Western Blot

A Western blot procedure was performed as described by Alves and collaborators [22]. In brief, 20 µg of protein from cell incubated with the different treatments was diluted in Laemmli sample buffer and β-mercaptoethanol and incubated for 10 min at 37 °C. After this, proteins were separated by size in a 15% polyacrylamide gel for approximately 120 min at 100 V. Then, proteins were electrotransferred from the gel onto a polyvinylidene difluoride membrane with a Trans-Blot^®^ Turbo^TM^ Transfer System (Bio-Rad, Hemel Hempstead, UK) for 7 min at 1.3 mA and 25 V. The membranes were incubated with Ponceau S staining solution (NZYtech, Lisbon, Portugal) to confirm proper protein transfer and detection was performed using a Bio-Rad ChemiDoc XRS (Bio-Rad, Hemel Hempstead, UK) and calculated with Image Lab^TM^ software (BioRad, Hemel Hempstead, UK). The membrane was then incubated with a blocking solution of 5% non-fat milk solution of Tris-buffered saline solution for 90 min at room temperature. Then, membranes were incubated overnight at 4 °C with goat anti-LDH (1:10,000, Abcam, Cambridge, UK), or goat anti-glucose transporter 2 (GLUT2) (1:5,000, Abcam, Cambridge, UK), or goat anti-monocarboxylate transporter 4 (MCT4) (1:5,000, Abcam, Cambridge, UK). The immune-reactive proteins were detected with goat anti-rabbit IgG-horseradish peroxidase (HRP) (1:5,000, Merck Millipore, Burlington, MA, USA). Membranes were incubated with WesternBright^TM^ ECL Chemiluminescent HRP Substrate (Advansta, Menlo Park, CA, USA) following the manufacturer’s instructions. WesternBright^TM^ ECL components were mixed in a 1:1 proportion to a final volume of 4 mL per membrane. Then, membranes were incubated with the solution for 2 min in a dark environment. Finally, the chemiluminescent signal was read with Bio-Rad ChemiDoc XRS (Bio-Rad, Hemel Hempstead, UK). Protein band quantification was performed using Image Lab^TM^ standard procedures. The value of band density was divided by the value of total protein previously acquired and normalized by dividing it by the mean of the control group and expressed in fold variation versus the control group.

### 2.10. Nuclear Magnetic Resonance Spectroscopy

The culture medium of each experimental group was collected before and after the treatment (0 and 24 h) for ^1^H-NMR analysis. The procedure used to obtain the spectra was similar to the one described by Alves and collaborators [23]. Briefly, spectra were obtained using samples of 220 µL run in a Varian 600 MHz spectrometer (Varian, Inc., Palo Alto, CA, USA), equipped with a 3 mm indirect detection probe (Varian, Inc., Palo Alto, CA, USA) at 25 °C. As an internal reference, sodium fumarate with a final concentration of 2 mM (singlet, 6.50 ppm) was used to quantify the metabolites in the extracellular medium: H1-α-glucose (duplet, 5.22 ppm), acetate (singlet, 1.9 ppm), alanine (duplet, 1.45 ppm), lactate (duplet, 1.33 ppm), 2-hydroxyisobutyrate (singlet, 1.35 ppm), 3-hydroxyisobutyrate (duplet, 1.08 ppm), and isobutyrate (duplet, 1.05 ppm). Spectra were manually phased, baseline corrected, and quantified by measuring the relative areas of ^1^H-NMR resonances using the curve fitting routine in the NUTSpro^TM^ spectral analysis program (Acorn NMR, Inc., Fremont, CA, USA). Results were normalized in relation to the quantities of the metabolites in the treatment medium before cell incubation and in relation to the number of seeded cells. The resulting values were expressed as metabolite consumption or production (µmol/million of cells).

### 2.11. Mitochondrial Membrane Potential

TM4 SCs were seeded and grown in black 96-well plates until reaching 70–90% confluence. Then, cells were incubated with the different treatment media. After the 24 h treatment, medium was removed and replaced by SC medium with the fluorescent probe 5–5′,6–6′-tetrachloro-1,1′,3,3′-tetraethylbenzimidazol-carbocyanine iodide (JC-1) at 2 µM and incubation was carried out for 30 min at 37 °C in a dark environment. After incubation, the medium was removed and standard SC medium was added. Excitation wavelengths of 485 nm and 535 nm and emission wavelengths of 530 and 590 nm were measured with a BioTek Synergy HT (BioTek, Winooski, VT, USA), pre-heated at 37 °C. Results were treated as the ratio of aggregates/monomers, divided by the mean of the control group and were expressed as a fold variation versus the control group.

### 2.12. Cellular Glycogen Content Assay

Quantification of glycogen content was performed using the colorimetric methodology described by Dubois and collaborators [24] with some modifications for the TM4 SCs utilized in this work. In brief, 5 million cells, from each treatment, were collected and incubated with potassium hydroxide (KOH) 0.5 M under agitation for 30 min at 4 °C. At midpoint, the cells were sonicated for 1 min. After incubation, the resulting solution was centrifuged at 16,000× *g* for 15 min at 4 °C. Then, phosphoric acid 10% was added to the supernatant. The samples were centrifuged at 16,000× *g* for 15 min at 4 °C. After, 100% ethanol was added to the supernatant and incubated overnight at 4 °C. On the following day, samples were centrifuged at 16,000× *g* for 15 min at 4 °C and the supernatant was discarded. The glycogen pellet was dissolved in lukewarm distilled water. After dissolution, 100 µL of the samples was treated with 100 µL of phenol at 6.5% followed by 500 µL of concentrated sulfuric acid. The samples were left to cool to room temperature. Different glycogen concentrations were used as standards for calibration and the absorbance of the samples was read at 490 nm using a BioTek Synergy HT (BioTek, Winooski, VT, USA). Results were normalized in relation to protein concentration of the sample and presented as fold variation versus the control group.

### 2.13. Oil Red O Staining

TM4 SC lipid droplet content was quantified using the Oil Red O staining technique [25]. Cells were seeded, grown, and treated for 24 h in 12-well plates with previously placed coverslips. When the desired confluence was reached, cells were washed with PBS with calcium ions (Ca^2+^) and magnesium ions (Mg^2+^) and fixed with a buffered solution of formalin 10% for 60 min at room temperature. After this, cells were incubated with isopropanol 60% for 5 min. Then, a solution of Oil Red Staining O^TM^ at 0.02% in water was filtrated and added to the fixed SCs for 20 min. After this period, the cells were washed multiple times with a solution of isopropanol at 50% and then with PBS, until all Oil Red O residues were removed. Then, the coverslips were removed from the 12-well plate and allowed to dry at 37 °C. Finally, the Oil Red O solution was dissolved from the lipid droplets with isopropanol 100% and then transferred to a 96-well plate for quantification through absorbance at 510 nm using a BioTek Synergy HT (BioTek, Winooski, VT, USA). The results were divided by the mean of the control group and expressed as fold variation versus the control group.

### 2.14. Statistical Analysis

Statistical significance between the samples from the different experimental groups was assessed by using an ordinary one-way ANOVA plus uncorrected Fisher’s LSD, with a single pooled variance test. Data from all experiments are presented as mean ± standard error of the mean (SEM) (N = 6; N refers to distinct passages of the TM4 cell line). Statistical analysis was performed using GraphPad Prism 6 (GraphPad Software, San Diego, CA, USA). Outliers were identified using the method of Grubbs with alpha equal to 0.2. The results were considered significant when *p* < 0.05. 

## 3. Results

### 3.1. GLP-1 Receptor and SGLT2 Are Expressed by Sertoli Cells

**Figure 1:**
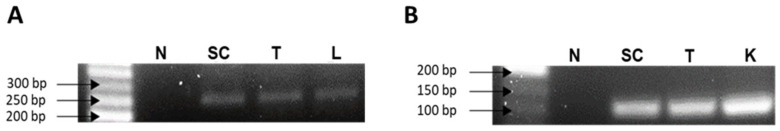
Identification of the expression of GLP-1 receptor (**A**) and SGLT2 (**B**) mRNA transcripts in TM4 Sertoli cells by reverse transcriptase polymerase chain reaction. Mouse lung, testis, and kidney total RNA were used as positive controls. Legend: N—Negative control; SC—TM4 Sertoli cells; T—Testis; L—Lung; K—Kidney.

The presence of GLP-1 receptor was previously reported in human SCs [26] and in mouse testes [27]. Likewise, the expression of SGLT2 has already been described in human testes [28]. Herein, we investigated whether the GLP-1 receptor and SGLT2 were expressed in TM4 mouse SCs using specific primer sets and obtained 253 bp and 104 bp amplicons, respectively, corresponding to the specific PCR amplification of the transcripts (Figure 1).

### 3.2. Exposure to Exenatide and/or Dapagliflozin Affects Neither Sertoli Cell Proliferation Nor Metabolic Viability

**Figure 2:**
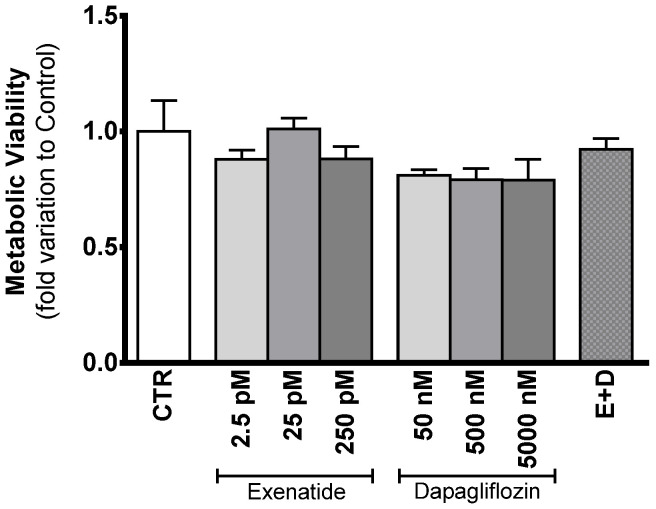
Evaluation of the cytotoxic profile of the different concentrations of exenatide and/or dapagliflozin on TM4 SCs. Results from the experimental groups of sub-pharmacological concentration (2.5 pM), pharmacological concentration (25 pM), and supra-pharmacological concentration (250 pM) of exenatide; sub-pharmacological (50 nM), pharmacological (500 nM), and supra-pharmacological concentration (5000 nM) of dapagliflozin; and the combined (E + D) treatment (25 pM exenatide plus 500 nM dapagliflozin); all in relation to the cells of the control group (CTR). The effect of the different treatments on metabolic viability was studied by MTT assay. The data are organized as pooled data of independent experiments and the results are expressed as mean ± SEM (N = 6 for each condition).

The data resulting from the assessment of the cytotoxic effect of the different drugs on cellular proliferation (SRB) did not show any difference between the different experimental groups (data not shown). Similarly, results obtained for the MTT assay did not show any variation in the metabolic viability between the cells exposed to the different treatments and those of the control group. The cells exposed to different concentrations of exenatide (2.5 pM, 25 pM, and 250 pM) exhibited a metabolic viability with a fold variation of 0.879 ± 0.133, 1.01 ± 0.048, and 0.881 ± 0.053 compared to control, respectively; the cells exposed to the different concentrations of dapagliflozin (50 nM, 500 nM, and 5000 nM) exhibited a metabolic viability with a fold variation of 0.810 ± 0.024, 0.791 ± 0.048 and 0.790 ± 0.089 compared to control, respectively; the cells exposed to the combined treatment of exenatide plus dapagliflozin exhibited a metabolic viability with a fold variation of 0.922 ± 0.0468 compared to control (Figure 2).

### 3.3. Combined Treatment of Exenatide plus Dapagliflozin Increases Glucose Consumption by Sertoli Cells

**Figure 3:**
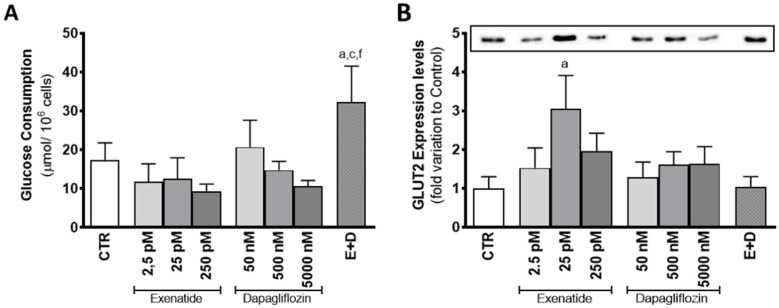
Effect of exenatide (2.5 pM, 25 pM, and 250 pM), dapagliflozin (50 nM, 500 nM, and 5000 nM), and their combined treatment (E + D) (25 pM exenatide plus 500 nM dapagliflozin) on glucose consumption (**A**) and glucose transporter 2 (GLUT2) expression levels (50 kDa) (**B**) in treated mouse SCs in relation to the ones of the control group (CTR). The data are organized as pooled data of independent experiments and the results are expressed as mean ± SEM (N = 6 for each condition). Significantly different results (*p* < 0.05) are indicated as: a—Relative to control (CTR); c—Relative to 25 pM exenatide; f—Relative to 500 nM dapagliflozin.

Mice SCs exposed to the combined treatment of exenatide and dapagliflozin had a glucose consumption of 32.2 ± 9.3 µmol/10^6^ cells, which was shown to be increased in comparison to that observed for control group cells (17.3 ± 4.5 µmol/10^6^ cells) and to the ones exposed to the pharmacological concentrations of dapagliflozin (14.7 ± 2.3 µmol/10^6^ cells) and exenatide (12.5 ± 5.4 µmol/10^6^ cells) alone. Moreover, cells exposed to exenatide at concentrations of 2.5 pM, 25 pM, and 250 pM did not exhibit any alteration in glucose consumption (11.7 ± 4.6; 12.5 ± 5.4; 9.2 ± 1.9 µmol/10^6^ cells, respectively) when compared with that of the cells of the control group. Exposure of SCs to the concentrations of 50 nM, 500 nM, and 5000 nM of dapagliflozin also did not change glucose consumption (20.7 ± 6.9; 14.7 ± 2.3; 10.6 ± 1.4 µmol/10^6^ cells, respectively) in relation to the cells of the control group (Figure 3A).

Exposure to dapagliflozin did not have any effect of mouse SC GLUT2 expression levels. Cells exposed to concentrations of 50 nM, 500 nM, and 5000 nM of dapagliflozin had GLUT2 expression levels with a fold variation of 1.29 ± 0.392; 1.61 ± 0.333; 1.63 ± 0.445 compared to control, respectively. On the other hand, mouse SCs exposed to pharmacological concentrations of exenatide (25 pM) showed increased levels of GLUT2 expression levels (3.05 ± 0.852 fold variation compared to control) in relation to the cells of the control group. However, expression levels of cells exposed to the combined treatment (1.04 ± 0.263 fold variation compared to control) were statistically equal to the ones of the control group, but different in comparison to the cells exposed to pharmacological concentrations of exenatide. Exposure to the other exenatide concentrations (2.5 pM and 250 pM) also did not have any effect on the expression levels of GLUT2 (1.52 ± 0.518; 1.96 ± 0.462 fold variation compared to control, respectively) in mouse SCs, when compared to the ones of the cells exposed to the control group medium (Figure 3B).

### 3.4. Combined Treatment of Exenatide plus Dapagliflozin Increased Lactate Production in Sertoli Cells

**Figure 4:**
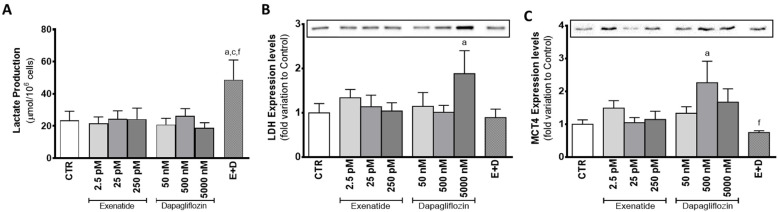
Effect of exenatide (2.5 pM, 25 pM, and 250 pM), dapagliflozin (50 nM, 500 nM, and 5000 nM), and their combined treatment (E + D) (25 pM exenatide plus 500 nM dapagliflozin) on lactate production (**A**), lactate dehydrogenase (LDH) expression levels (37 kDa) (**B**), and monocarboxylate transporter 4 (MCT4) expression levels (50 kDa) (**C**) in treated mouse Sertoli cells in relation to the ones of the control group (CTR). The data are organized as pooled data of independent experiments and the results are expressed as mean ± SEM (N = 6 for each condition). Significantly different results (*p* < 0.05) are indicated as: a—Relative to control (CTR); c—Relative to 25 pM exenatide; f—Relative to 500 nM dapagliflozin.

Mouse SCs exposed to the combined treatment of pharmacological concentrations of dapagliflozin and exenatide showed enhanced lactate production (48.7 ± 12.3 µmol/10^6^ cells) in comparison to the ones of the control group (23.4 ± 5.7 µmol/10^6^ cells) and to the ones exposed to the pharmacological concentrations of dapagliflozin and exenatide separately (26.3 ± 4.5 and 24.5 ± 5.0 µmol/10^6^ cells, respectively). However, in comparison to the cells from the control group, cells exposed to dapagliflozin (50 nM, 500 nM, and 5000 nM) did not have significantly different lactate production (20.9 ± 3.9; 26.3 ± 4.5; 18.8 ± 3.2 µmol/10^6^ cells, respectively). Likewise, cells exposed to exenatide (2.5 pM, 25 pM, and 250 pM) also did not exhibit any statistically alteration in lactate production (21.7 ± 3.9; 24.4 ± 5.0; 24.2 ± 6.9 µmol/10^6^ cells, respectively) in comparison to SCs of the control group (Figure 4A).

Mouse SCs exposed to pharmacological concentrations of dapagliflozin, which exhibited an increase in lactate production, did not present any alteration in LDH expression levels (1.01 ± 0.16 fold variation compared to control) in relation to the cells of the control group. Moreover, sub-pharmacological concentrations of dapagliflozin (50 nM) also did not have any effect on LDH expression levels (1.15 ± 0.31 fold variation to control), when compared to the cells of the control group. However, SCs exposed to supra-pharmacological concentrations of dapagliflozin (5000 nM) showed increased expression levels of LDH (1.88 ± 0.52 fold variation compared to control) when compared to the cells of the control group. Those exposed to the different concentrations of exenatide (2.5 pM, 25 pM, and 250 pM) had no alteration in LDH expression levels (1.34 ± 0.19; 1.14 ± 0.26; 1.04 ± 0.19 fold variation compared to control, respectively) (Figure 4B). Furthermore, SCs of the different experimental groups did not show any alteration in LDH activity (data not shown) in relation to the cells of the control group.

Dapagliflozin (at a concentration of 500 nM) was able to increase the expression levels of MCT4 (2.26 ± 0.65 fold variation compared to control) in SCs, as compared to cells from the control group. However, the combined treatment of exenatide plus dapagliflozin, which was able to increase lactate production by SCs, was not capable of significantly increasing the expression levels of MCT4 by these cells (0.751 ± 0.053 fold variation compared to control), although it was statistically lower than the MCT4 expression levels observed in the cells exposed to the pharmacological concentrations of dapagliflozin. The cells exposed to 50 nM and 5000 nM dapagliflozin had MCT4 expression levels (1.34 ± 0.20; 1.68 ± 0.40 fold variation compared to control, respectively) which were not different from the control group. Similarly, exposure to exenatide (2.5 pM, 25 pM, 250 pM) did not alter the expression levels of MCT4 (1.49 ± 0.23; 1.05 ± 0.16; 1.15 ± 0.25 fold variation compared to control, respectively) in SCs, when compared to that of cells from the control group (Figure 4C).

### 3.5. Combined Exposure to Pharmacological Concentrations of Exenatide and Dapagliflozin Increased Alanine Production and Restored Acetate Production by Sertoli Cells

**Figure 5:**
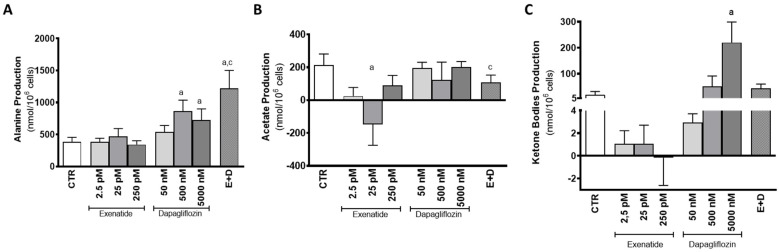
Effect of exenatide (2.5 pM, 25 pM, and 250 pM), dapagliflozin (50 nM, 500 nM, and 5000 nM), and their combined treatment (E + D) (25 pM exenatide plus 500 nM dapagliflozin) on alanine (**A**), acetate (**B**), and ketone body production (**C**) by treated mouse Sertoli cells in relation to the ones of the control group (CTR). The data are organized as pooled data of independent experiments and the results are expressed as mean ± SEM (N = 6 for each condition). Significantly different results (*p* < 0.05) are indicated as: a—Relative to control (CTR); c—Relative to 25 pM exenatide.

Mouse SCs exposed to pharmacological and supra-pharmacological concentrations of dapagliflozin (500 nM and 5000 nM) and the cells exposed to the combined treatment produced higher amounts of alanine (863 ± 174; 728 ± 174; 1218 ± 284 nmol/10^6^ cells, respectively) in relation to the cells from the control group (387 ± 68 nmol/10^6^ cells). On the other hand, cells exposed to 50 nM of dapagliflozin did not show any alteration in the production of alanine (539 ± 102 nmol/10^6^ cells). SCs exposed to the different concentrations of exenatide (2.5 pM, 25 pM, 250 pM) also did not exhibit any alteration in alanine production (383 ± 60; 471 ± 122; 342 ± 60 nmol/10^6^ cells, respectively), when compared with the cells from the control group. Additionally, it is worth noting that cells exposed to the combined treatment of exenatide plus dapagliflozin had significantly higher production of alanine when compared to the cells exposed to pharmacological concentrations of exenatide (25 pM) (Figure 5A).

Mouse SCs exposed to pharmacological concentrations of exenatide (25 pM) consumed acetate (147 ± 127 nmol/10^6^ cells) in contrast to the cells of the control group that produced acetate (214.6 ± 67.8 nmol/10^6^ cells). Cells exposed to the other concentrations of exenatide (2.5 pM and 250 pM) also exhibited acetate production (25.2 ± 52.3; and 89.9 ± 60.5 nmol/10^6^ cells, respectively), which was statistically identical to the production from the cells of the control group. Cells exposed to the combined treatment of pharmacological concentrations of exenatide plus dapagliflozin exhibited an acetate production of 108 ± 45 µmol/10^6^ cells, which is also statistically equal to that of cells from the control group, but different when compared to the cells exposed to pharmacological concentrations of exenatide. Moreover, cells exposed to the various concentrations of dapagliflozin (50 nM, 500 nM, and 5000 nM) had a similar acetate production (195.9 ± 34.9; 122.4 ± 109.3; 202.1 ± 33.4 nmol/10^6^ cells, respectively) in comparison to that of cells from the control group (Figure 5B).

Dapagliflozin, however, when in the supra-pharmacological concentration of 5000 nM, was able to increase the production of ketone bodies by mouse SCs (220 ± 79 nmol/10^6^ cells) in comparison to the production observed by the cells of the control group (18.4 ± 13.1 nmol/10^6^ cells). The remaining dapagliflozin-exposed experimental groups (50 nM and 500 nM) presented no alteration in ketone body production (51.4 ± 39.7; 1.61 ± 0.99 nmol/10^6^ cells), as well as those exposed to 2.5 pM, 25 pM, and 250 pM of exenatide (1.07 ± 1.15; 1.05 ± 1.65; −0.169 ± 2.45 nmol/10^6^ cells) (Figure 5C).

### 3.6. Sub-Pharmacological Concentrations of Exenatide Decreased Mitochondrial Membrane Potential in Sertoli Cells

**Figure 6:**
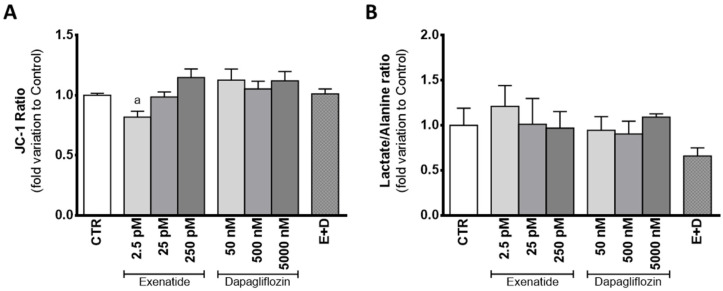
Effect of exenatide (2.5 pM, 25 pM, and 250 pM), dapagliflozin (50 nM, 500 nM, and 5000 nM), and their combined treatment (E + D) (25 pM exenatide plus 500 nM dapagliflozin) on mitochondrial membrane potential measured by JC-1 ratio (**A**) and lactate/alanine ratio (**B**) of treated mouse Sertoli cells in relation to the ones of the control group (CTR). The data are organized as pooled data of independent experiments and the results are expressed as mean ± SEM (N = 6 for each condition). Significantly different results (*p* < 0.05) are indicated as: a—Relative to the control group (CTR).

SC exposure to sub-pharmacological concentrations of exenatide (2.5 pM) showed a decreased JC-1 ratio (0.819 ± 0.047 fold variation compared to control) in relation to the cells of the control group, which means that exposure of mouse SCs to this exenatide concentration results in decreased mitochondrial membrane potential. However, at concentrations of 25 pM and 250 pM, exenatide did not have an effect on the mitochondrial membrane potential (0.987 ± 0.049; 1.15 ± 0.072 fold variation compared to control, respectively) in comparison to cells from the control group. Cells exposed to dapagliflozin (50 nM, 500 nM, and 5000 nM) also did not show any alteration in mitochondrial membrane potential (1.13 ± 0.09; 1.05 ± 0.06; 1.12 ± 0.08 fold variation compared to control, respectively), when compared to that of cells from the control group. Similarly, mouse SCs exposed to combined treatment of exenatide plus dapagliflozin also did not show any alteration in mitochondrial membrane potential (1.01 ± 0.04 fold variation compared to control) when compared to that of cells from the control group (Figure 6A).

The mild depolarization observed in the cells exposed to the sub-pharmacological concentration of exenatide (2.5 pM) could be linked to the lactate/alanine ratio, an indirect marker of the redox state of the cells. Still, there was no difference between the ratio of lactate/alanine produced by the cells exposed to the sub-pharmacological concentration of exenatide (1.21 ± 0.23 fold variation compared to control) and the ones from the control group. In fact, the lactate/alanine ratio of the SCs was not altered by any of the experimental conditions assayed, when compared to the one observed in the cells from the control group. The cells exposed to exenatide (25 pM and 250 pM) had a lactate/alanine ratio with a 1.01 ± 0.29; 0.969 ± 0.184 fold variation compared to control, respectively, whereas the ones exposed to dapagliflozin (50 nM, 500 nM, and 5000 nM) had a lactate/alanine ratio with a 0.945 ± 0.151; 0.905 ± 0.141; 1.09 ± 0.035 fold variation to control, respectively. Finally, combined exposure to dapagliflozin plus exenatide resulted in a lactate/alanine ratio with a 0.659 ± 0.090 fold variation compared to control (Figure 6B).

### 3.7. Exposure to Supra-Pharmacological Concentration of Dapagliflozin Decreases Lipid Accumulation in Sertoli Cells

**Figure 7:**
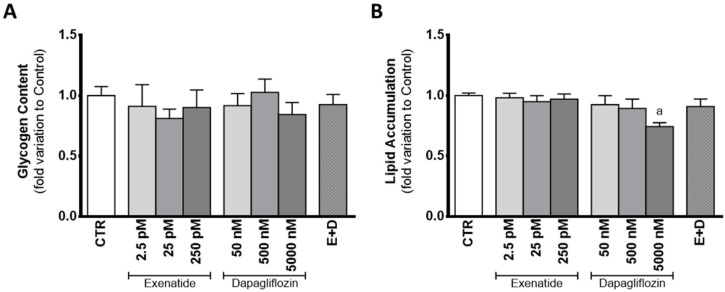
Effect of exenatide (2.5 pM, 25 pM, and 250 pM), dapagliflozin (50 nM, 500 nM, and 5000 nM), and their combined treatment (E + D) (25 pM exenatide plus 500 nM dapagliflozin) on glycogen content (**A**), and in lipid accumulation (**B**) of treated mouse Sertoli cells in relation to the ones of the control group (CTR). The data are organized as pooled data of independent experiments and the results are expressed as mean ± SEM (N = 6 for each condition). Significantly different results (*p* < 0.05) are indicated as: a—Relative to control (CTR).

In the presence of dapagliflozin, mouse SC glycogen content was statistically equal to that observed in the cells from the control group. When exposed to dapagliflozin (50 nM, 500 nM, and 5000 nM), SCs showed a glycogen content with a 0.918 ± 0.099; 1.03 ± 0.11; 0.844 ± 0.099 fold variation compared to control, respectively. Similarly, cells exposed to exenatide (2.5 pM, 25 pM, and 250 pM) did not differ in glycogen content (0.911 ± 0.179; 0.812 ± 0.077; 0.901 ± 0.146 fold variation compared to control, respectively), when compared to that of cells from the control group. Glycogen content of SCs exposed to the combined treatment of exenatide plus dapagliflozin had a 0.925 ± 0.084 fold variation compared to control, which was not different from that of cells of the control group (Figure 7A).

Despite no difference found in lipid content of cells exposed to pharmacological concentrations of dapagliflozin (0.894 ± 0.077 fold variation compared to control) when compared to that of cells of the control group, cells exposed to supra-pharmacological concentrations of dapagliflozin showed decreased lipid content (0.743 ± 0.033 fold variation compared to control). Cells exposed to sub-pharmacological concentration of dapagliflozin and those exposed to exenatide (2.5 pM, 25 pM, and 250 pM) had a lipid content (0.925 ± 0.074, 0.982 ± 0.895; 0.949 ± 0.049; 0.970 ± 0.044 fold variation compared to control, respectively) similar to that of the cells of the control group. The results obtained for the lipid content of SCs exposed to combined treatment of exenatide plus dapagliflozin (0.909 ± 0.063 fold variation compared to control) were also not different in comparison to the control group (Figure 7B).

## 4. Discussion

The incidence of metabolic diseases, such as type 2 DM and obesity, has been increasing worldwide and estimations in recent studies indicate that the tendency is to be aggravated further [29]. Both diseases have a profound effect on lifestyle and life expectancy of the individual. Pharmaceutical companies are constantly trying new approaches for developing new pharmacological agents to control the adverse effects that metabolic diseases have. Pharmacological agents are developed to ensure that their effect is optimized on their target cells and systems. However, they can impact other unwanted targets, despite the effort to minimize off-target adverse effects. This was previously described with several anti-diabetic drugs, where, for instance, it was noticed that they were able to modulate the metabolism of testicular cells [16]. Nevertheless, as far as we know, no study has been performed regarding the effect of the drugs exenatide and dapagliflozin on SC physiology and metabolism. Thus, the objective of this work was to investigate whether the exposure to each one of these two drugs modulates SC glucose metabolism and if the combination therapy of both drugs might be potentially safe for male reproductive function, using an in vitro approach.

**Figure 8:**
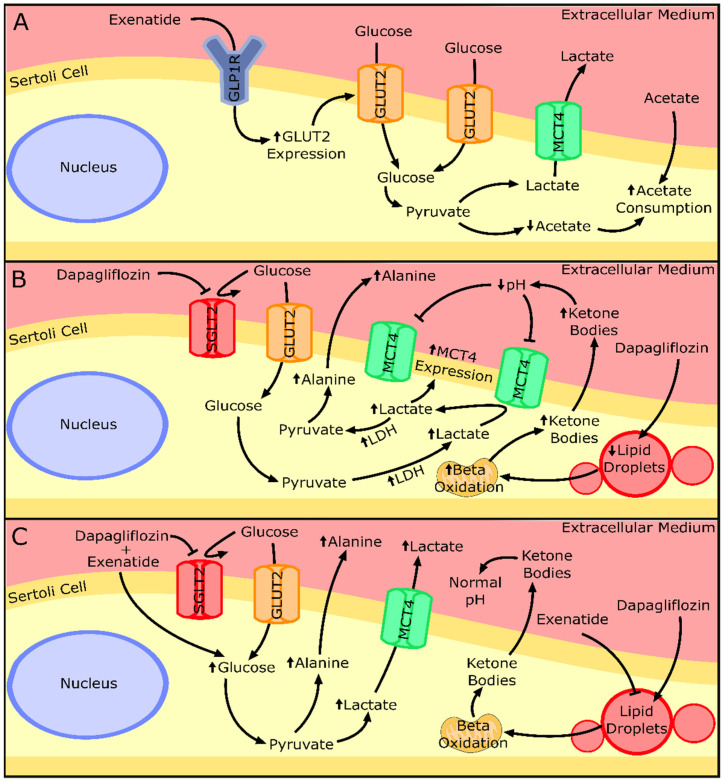
Summary of the effects of the exenatide or dapagliflozin and their combination on the metabolism and secretion of key metabolites by Sertoli cells. (**A**) Sertoli cells exposed to exenatide treatment presented increased glucose transporter 2 (GLUT2) expression and acetate consumption. (**B**) Sertoli cells exposed to dapagliflozin treatment presented overexpression of monocarboxylate transporter 4 (MCT4) and lactate dehydrogenase (LDH). A decrease in lipid droplets and an increase in lipid β-oxidation may be the origin of the overproduction of ketone bodies that decrease the extra-cellular pH, which inhibits lactate secretion. Lactate can be converted into pyruvate and, after, to alanine, explaining its increased secretion by Sertoli cells. (**C**) Combined treatment of exenatide and dapagliflozin shown to increase the glycolytic flux of the Sertoli cells, resulting in increased secretion of lactate and alanine. The presence of exenatide could restore ketone body production and allow lactate secretion. Upward or downward arrows represent overexpression/underexpression of proteins or higher/lower concentration of metabolites, respectively, in relation to the control group. Legend: GLP1R—Glucagon-like peptide 1 receptor; SGLT2—Sodium/glucose co-transporter 2.

The results of cytotoxic assays performed in this study corroborate the results of a previous clinical trial in demonstrating a lack of detrimental effects of the tested drugs on SCs [10]. Our results also showed the ability of exenatide to maintain a normal glucose consumption by mouse SCs. In a previous study, GLP-1 at post-prandial concentrations (10 pM) decreased glucose consumption by human SCs, after 6 h of exposure [26]. The discrepancy between these published results and our own could reflect the different sensitivities of the receptor towards the two peptides (GLP-1 and exenatide) and/or the different incubation times [30]. It is worth noting that exenatide has a half-life time from 20- to 30-fold higher when compared to the one of GLP-1 [30]. This could also be related to the increased expression of GLUT2 observed when mouse SCs were exposed to pharmacological concentrations of exenatide (25 pM). In order to reverse the hypothetical decrease in glucose consumption associated with GLP-1 agonists, murine SCs exposed to exenatide might resort to increasing GLUT2 levels. GLUT2 has been described as one of the most predominant glucose transporters in SCs [31] and studies have already highlighted that its expression influences the glucose consumption of this cell type [4,17]. Thus, the adaptative abilities and metabolic plasticity of SCs to different external conditions [17,26] are once more emphasized in this experimental design. In fact, our results indicated that mouse SCs were able to maintain lactate and alanine production in the presence of exenatide, independent of the concentration used, when compared to that of the cells from the control group. However, the other monocarboxylate (acetate) usually secreted in high amounts to the extracellular medium by SCs [32,33] was consumed when these cells were exposed to the pharmacological concentration of exenatide (25 pM) (Figure 8A). Still, the model used in our experiment (TM4 SCs) exhibited an acetate production of almost 1000-fold lower than that reported for human SCs [32]. Thus, these low concentrations of acetate produced by the TM4 SCs of the control group are probably linked to a cell-specific variation observed in all our experimental groups. Nonetheless, this could also mean that the metabolic function of SCs might be compromised, because SCs are known to secrete high amounts of acetate to the extracellular medium [32] to maintain a high rate of lipid synthesis necessary for the formation and maturation of new germ cells [33].

The cells exposed to sub-pharmacological concentrations of exenatide (2.5 pM) showed decreased mitochondrial membrane polarization, which could be due to the destabilization of the redox state of the cells. To study that hypothesis, we analyzed one indirect indicator of the redox state of the cell: the ratio of lactate/alanine [17]. Despite not reaching statistical significance, the results of the lactate/alanine ratio show a tendency to increase with lower concentrations of exenatide, which is the opposite of what is seen in the results of the JC-1 ratio, which increases with higher concentrations of exenatide. This could indicate that the depolarization of the mitochondrial membrane potential can be provoked by a change in the redox state of the SCs in lower concentrations of exenatide (2.5 pM). The literature on this subject points to the ability of exenatide to protect mitochondrial function from oxidative damage [34,35]. However, in these studies, the dosage of exenatide was equal to or higher than the pharmacological concentration of exenatide tested in our work (25 pM), which did maintain the mitochondrial membrane potential in mouse SCs.

The study of the mouse SCs’ metabolic profile when exposed to dapagliflozin showed no difference in glucose consumption, despite the main pharmacological property of this compound being the inhibition of a transporter of glucose (SGLT2). This highlights the secondary role of this transporter in relation to GLUTs in consuming glucose in SCs. Moreover, even without increasing glucose consumption, exposure to pharmacological (500 nM) and supra-pharmacological (5000 nM) concentrations of dapagliflozin enhanced the production of alanine by mouse SCs. Alanine production is regarded as an indicator of increased glycolytic flux in cells with a highly glycolytic metabolism [36] such as the SCs. Enhanced glycolytic flux should be accompanied by an increase in lactate production since that is the prioritized pathway by healthy SCs [37]. However, our results showed no alteration in lactate production by SCs exposed to the dapagliflozin concentrations, despite the increased expression levels of MCT4 and LDH observed in cells exposed to pharmacological and supra-pharmacological concentrations of dapagliflozin, respectively. These results could be explained by the lactate accumulation inside of the cell, but this is unlikely since the transport direction of MCT4 is determined by the net driving force for lactate and proton H^+^ [38]. Taking this into consideration, if the extracellular medium has a low pH, the SCs may be incapable of exporting lactate [39]. The production of ketonic bodies by the SCs [40], which was described to be increased in adipocytes under dapagliflozin treatment (0.02 mg/mL) [41] and in hyperglycemic conditions [42,43,44], could also decrease extracellular medium pH. Indeed, our results show that supra-pharmacological concentrations of dapagliflozin increase the secretion of ketone bodies by SCs. Thus, increased ketonic body production decreases extracellular pH [45] in the in vitro conditions of this work, decreasing the net driving force for the proton H^+^ and possibly diminishing lactate secretion through MCT4. In this case, the enhanced LDH expression levels observed at higher dapagliflozin levels (5000 nM) could indicate the decrease in the hypothetical cytosolic lactate accumulation, since LDH is also able to metabolize lactate into pyruvate (reverse reaction). The decrease in the cellular lipidic reserves noticed with the supra-pharmacological concentration of dapagliflozin strengthens the theory of SCs’ ketonic body overproduction. Moreover, it also evidences the anti-obesogenic ability of dapagliflozin at a cellular level, which was already described in diabetic patients under dapagliflozin treatment (5 mg/day) that decreased their liver fat accumulation during a 6-month treatment [46]. These results also highlight the role of supra-pharmacological concentrations of dapagliflozin in the decrease in intracellular fat deposits in SCs (Figure 8B).

Lundkvist and co-workers examined the anti-obesogenic potential of the combined treatment of dapagliflozin (10 mg/day) plus exenatide (2 mg/week) and their results showed an additive effect in the combined therapy, since the weight loss was greater than with each monotherapy [12]. This ability seems to be due to their independent mechanism of action and, at a cellular level, as far as our results show, this seems to also be the case. The presence of both drugs in the medium increased glucose consumption, lactate, and alanine production by mouse SCs in what seems to be an additive manner in relation to the ones exposed to the monotherapy with a pharmacological concentration. One would assume that these results point to the ability of the treatment to further enhance the glycolytic pathway of the SCs. To support this possibility, we further investigate other carbon sources such as glycogen cellular content and lipid accumulation. It has been described that SCs increase the glycogen reserves in hyperglycemic conditions [47] and are able to mobilize them when in hypoglycemic conditions [48,49]. In addition, it is widely described that SCs can also perform lipolysis and mobilize reserves when needed [49,50]. However, our results showed that this was not the case in the mouse SCs exposed to the combined treatment of dapagliflozin and exenatide. With no mobilization of glycogen or lipid reserves, the increase in glucose consumption is the only carbon source that we were able to identify, which could be used to produce the extra lactate and alanine in relation to the control group. Although our results showed that LDH expression and activity were maintained in these conditions, it is known that this enzyme can catalyze not only the forward reaction but also the reversible reaction. This could mean that LDH could be prioritizing lactate synthesis when cells are exposed to both drugs simultaneously. Moreover, it has already been described that exenatide was able to reverse lipid-induced cellular dysfunction in epithelial cells [51]. A similar phenomenon could have happened in our experiment, decreasing the possible ketone body production by the dapagliflozin and enabling the lactate to be secreted to the extracellular medium. Taking these results into consideration, the combined treatment seems to increase the glucose consumption, enhancing the glycolytic pathway, and with an additive effect in lactate and alanine production. However, a possible complementary ability of exenatide in decreasing lipolysis-induced metabolic alterations provoked by dapagliflozin was also observed in cells exposed to both drugs simultaneously (Figure 8C).

With this work, we were able to determine that pharmacological concentrations of exenatide maintained glucose consumption and lactate production, while pharmacological concentrations of dapagliflozin further increased alanine production, as well as MCT4 expression levels, which is an important contributor for lactate secretion for developing germ cells. The results from the monotherapies did not show any effects on major key spermatogenic metabolites (such as lactate), which can indicate that neither drug disrupts nor promotes the nutritional support of spermatogenesis. However, the combined treatment of pharmacological concentrations of both drugs demonstrated an additive effect on SCs’ lactate and alanine production as well as glucose consumption, when compared to the monotherapies. This shows that the independent action of the drugs can enhance the glycolytic pathway in these cells. This combined exposure was able to enhance the secretion of lactate by SCs, which is crucial for the proper development of spermatogenesis. Therefore, these novel findings suggest that combined treatment of dapagliflozin plus exenatide might be an interesting approach for the treatment of diabetic and obese males of reproductive age.

## Figures and Tables

**Table 1:** Genes, oligonucleotide sequence, and respective conditions for polymerase chain reaction amplification of GLP-1 receptor and SGLT2 transcripts.

mRNA	Sequence (5′-3′)	AT (°C)	Amplicon (bp)	Cycles
GLP-1 receptor NM_021332.2	Sense: GATGCTGCCCTCAAGTGGAT Anti-sense: TAACGAACAGCAGCGGAACT	61	253	37
SGLT2 NM_133254.3	Sense: ATGGAGCAACACGTAGAGGC Anti-sense: ATGACCAGCAGGAAATAGGCA	63	104	35

Legend: AT—Annealing temperature; GLP-1—Glucagon-like peptide 1; SGLT2—Sodium/glucose cotransporter 2.

## Data Availability

Not applicable.
